# Anisotropic Growth of Filamentous Fungi in Wood Hydrogel Composites Increases Mechanical Properties

**DOI:** 10.1021/acsabm.5c00374

**Published:** 2025-06-06

**Authors:** Ciatta Wobill, Ziyu Zhang, Peter Fischer, Patrick A. Rühs

**Affiliations:** Institute of Food, Nutrition, and Health, ETH Zürich, 8092 Zürich, Switzerland

**Keywords:** mycelium, tensile test, anisotropy, delignified wood, composite material, engineered living materials

## Abstract

There is a rising demand for sustainable, biodegradable, and robust materials in response to growing environmental concerns. Here, we propose using delignified wood as a scaffold for fungal proliferation to obtain wood–fungi composites. The delignification process preserves the fiber directionality inherent to natural wood, enabling fungi to grow along these fibers, enhancing the composites’ mechanical properties, and promoting anisotropic fungal growth. The delignified wood was used as a scaffold for the growth of *Aspergillus oryzae* and *Rhizopus oligosporus*. Both wood–fungi composites exhibited a higher mechanical strength after fungal proliferation. We used balsa, poplar, and spruce as wood to demonstrate the effects of varying wood architectures. Even though the tensile strengths of all three wood scaffolds were not significantly different, wood scaffolds with lower densities promoted fungal growth. Increasing agar and glucose concentrations were found to significantly enhance tensile strength and Young’s modulus. The tensile strength and Young’s modulus of wood scaffolds increased from 10^1^ kPa to nearly 10^3^ kPa and 10^–3^ GPa to nearly 10^–1^ GPa, respectively. These results highlight the versatile nature of delignified wood as a platform for fungal growth. It offers tunable properties that can be optimized for various applications in composite manufacturing.

## Graphical abstract

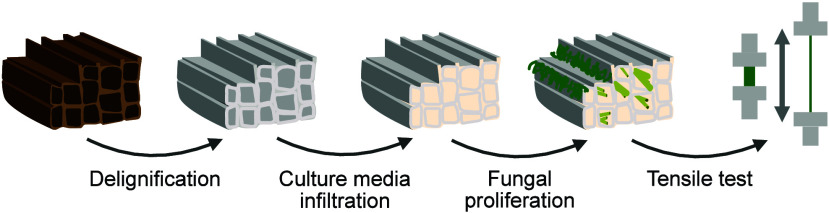

## Introduction

Engineered living materials (ELMs) are composed of living cells integrated within a matrix, with the living cells capable of generating and modifying the matrix themselves.^1–4^ The matrix is specifically fine-tuned to support the living cells, allowing them to perform functions such as metabolite production, stimulus-response, and the exhibition of desired mechanical properties.^2,3^ Typically, bacteria and fungi are utilized due to their metabolic and structural characteristics, particularly suited for ELMs.^5,6^ The microbes contribute the necessary enzymes, exopolysaccharides, and metabolites, which in conjunction with the host matrix result in materials with tailored functional and mechanical properties.^3^ Among these, filamentous fungi demonstrate advantageous qualities for developing such materials.^6,7^ They comprise a large-scale network called mycelium, consisting of intertwined filaments called hyphae.^8^ Due to their rapid growth, they can occupy large spaces within a short time frame.^2,9^ They also exhibit self-healing properties, allowing them to regenerate after damage.^1,10^ Most importantly, they can adapt to their environment by adjusting to varying nutrient concentrations, viscoelasticity, and chemical composition of the culture media, enabling the development of smart, functional, and mechanically robust materials.^6,11–13^ However, most material-microbe hybrids and composites are made from purely agar-based materials, allowing for fungal growth but not providing robust and hollow growth scaffolds for fungi.^10^

On homogeneous culture media, such as hydrogel-based nutrient media, fungal growth is isotropic, following a radial direction from the inoculation point.^14^ When mycelium is confined, either between plant cells or within inert materials like plastic, the growth becomes directed and exhibits anisotropy.^15,16^ To utilize and enhance the fibrous structure of fungi, employing anisotropic fungal growth could further improve the mechanical properties. Anisotropic material possesses high tensile strength and fracture toughness along the fiber direction.^17^ A study conducted by Kozicki et al.^18^ demonstrated directed mycelial growth on integrated circuits through galvanotropism.^19^ Gruler et al.^20^ used similar approaches in an electronic field to achieve directed fungal growth, resulting in the germ tube growing toward the anode. However, these techniques are neither sustainable nor feasible for the development of ELMs. To promote anisotropic fungal growth sustainably, it is essential to direct the fungal growth using a robust material that cannot or can only slowly be degraded by the fungus while providing mechanical features beyond agar.

Wood forms by nature a robust and anisotropic fiber material, that, following delignification, forms a porous scaffold with aligned fibers.^21^ Delignified wood is an ideal, biodegradable platform for developing tunable materials with desirable mechanical properties.^22^ Additional postprocessing, such as densification can lead to even mechanically stronger material.^21,23,24^ Infiltrating the wood with epoxy or mineralized hydrogel results in a material with very high mechanical strength, for applications in building or bone research, whereas infiltration with hydrogels results in a lower strength material for soft materials application.^25–27^ In soft materials application, the selected hydrogel can be cross-linked to enhance mechanical strength to meet specific requirements or optimal growth conditions for cells and microbes.^25,26^ Moreover, increasing the concentration of hydrogels is expected to improve the mechanical properties of the composite material.^28^ In conclusion, delignified wood is a versatile platform for composite manufacturing, offering a range of material properties from stiff to soft and flexible. The delignification process preserves the fiber directionality of natural wood.^23^ Such scaffolds can be impregnated with nutrients and fungi, serving as an excellent scaffold for guiding fungal growth. We drew inspiration from nature, where fungi naturally thrive on plant and wood material, observing how fungi align with plant cells and grow in the spaces between them.^29^ In this context, a delignified wood hydrogel composite emerged as an ideal candidate. We examined the fungal growth within delignified wood-hydrogel composites and the effects of wood type and hydrogel culture media for two different fungi, *Aspergillus oryzae* and *Rhizopus oligosporus*. We used tensile tests to determine the effects of fungal proliferation, agar impregnation, and nutrient concentration on the final mechanical properties.

## Materials and Methods

### Wood Hydrogel Composite Preparation and Fungal Inoculation

Wood veneers cut tangential to the tree (spruce, balsa, poplar) were delignified accordingly to Frey et al.^22^ In short, wood veneers were cut into 4 × 2 × 0.14 cm pieces and soaked in a 1:1 ratio bath of hydrogen peroxide (Fisher Scientific, Netherlands) and acetic acid (Supelco, Germany) overnight. The bath was heated to 80 °C for 6 h, and then the delignified wood pieces were removed and washed with distilled water until a pH of 6 was reached. The culture media for infiltration consisted of 2 wt % malt extract (Morga, Switzerland) and 0.2 wt % yeast extract (Sigma-Aldrich, France), both solubilized in distilled water. If indicated, 0.5–4 wt % agar (Morga, Switzerland) or 5 wt % glucose (VWR Chemicals, USA) were added. The delignified wood pieces were soaked overnight in the culture media at 60 °C and subsequently subjected to a vacuum treatment for 1 h. Afterward, the vacuum was released and the delignified wood pieces were removed from the bath and UV-treated for 30 min per side for disinfection.

*Aspergillus oryzae* and *Rhizopus oligosporus* were used for all experiments. For both, we purchased a commercial spore powder (Kawashimaya Koji Starter for Shoyu, Hishiroku Shop Kyoto, Japan or Tempeh Spores, fermentation culture, Austria) and purified it to one phenotype over 5 incubation cycles. Then, the spores were harvested and used for further growth trials. The delignified wood pieces were inoculated with a piece of mycelium no older than 7 days. The piece of mycelium was put next to the longitudinal side of the wood so the fungus could grow along the fiber direction of the wood.

### Radial Growth Measurements of Solid Cultures

The radial growth rate was determined in Petri dishes filled with agar culture media. A spore solution in distilled water was prepared and pipetted into the middle of the dish. The plates were incubated at 30 °C and with 95% relative humidity. The radial growth was measured every 24 h for 7 days, resulting in the culture diameter in mm per day.

### Sample Characterization

#### Fourier-Transform Infrared Spectroscopy

Fourier-transform infrared spectroscopy (FTIR) was used to evaluate the efficacy of the delignification process. The FTIR spectra were obtained by employing a Varian 640 FTIR spectrometer (Varian, Austria) equipped with a Golden Gate diamond ATR. Samples were scanned in the range from 4000 to 600 cm^–1^ at a resolution of 4 cm^–1^ and at room temperature. Each spectrum was sampled with 64 scans, and the final analyzed spectrum was averaged with three independently acquired spectra.

#### Scanning Electron Microscopy

The microstructural morphologies of the samples were visualized by a Scanning Electron Microscope (SEM). Small rectangular test pieces about 1 mm wide and 10 mm long were cut out of the wood hydrogel composites. Sample processing was done in a PELCO BioWave, Pro+ microwave system (Ted Pella, USA), where a microwave-assisted fixation was performed followed by a dehydration procedure. Imaging was done at an accelerating voltage of 2 lV (Merlin, Zeiss, Germany); SE-inlens and Everhart.Thorley (ET) SE-Signals were recorded at a working distance of around 5 mm. Detailed information on the fixation and mounting can be found in the Supporting Information.

#### Mechanical Analysis

Tensile tests were performed on an Anton Paar MCR 702 rheometer (Anton Paar, Austria) equipped with a linear motor. The samples were cut into a rectangular shape of about 6 mm in width and 40 mm in length and fixed to a rectangular tensile fixture (U-SRF5, L-SRF5/LD/TS, Anton Paar, Austria). The pieces were extended along the fiber direction with a constant extensional velocity of 10 μm/s.

#### Density Measurements

The density of the wood and delignified wood composites was measured gravimetrically by dividing the weight by the volume.

### Statistical Analysis

Statistical analysis was conducted using RStudio (R 4 with RStudio 2021 4.4.2/2024.12.0), with an alpha level of 0.05 set as the threshold for significance. Young’s moduli and maximum stresses were transformed into a logarithmic scale for subsequent analysis to enhance the accuracy of the analysis by stabilizing variance and making the data more normally distributed. For ANOVA, a linear model was constructed to evaluate the effects of different factors on the mechanical strength of wood composites. Multiple comparisons and posthoc tests were performed to further investigate the differences between species treatment groups after overall significant results were identified through ANOVA. Specifically, Tukey’s Honestly Significant Difference (Tukey HSD) test was used to conduct pairwise comparisons among the groups. *t* tests or Welch’s tests were performed to pairwise compare single means in Origin Pro 2024 10.1.0.178 (OriginLab Corporation). The notations for the significance of the *p*-values are between 0 and 0.001 “***”, between 0.001 and 0.01 “**”, between 0.01 and 0.05 “*”, between 0.05 and 0.1 “.”, and between 0.1 and 1 “ ”.

## Results and Discussion

Delignified wood was utilized as a scaffold for fungal proliferation. The wood was delignified and infiltrated with culture media with and without agar (Figure 1). Following successful delignification and infiltration, the wood scaffolds were inoculated with fungal mycelium, *Aspergillus oryzae* and *Rhizopus oligosporus*, and incubated. The mechanical properties of the wood scaffolds, wood scaffolds with agar, and wood–fungi composites were measured by tensile testing.

**Figure 1:**
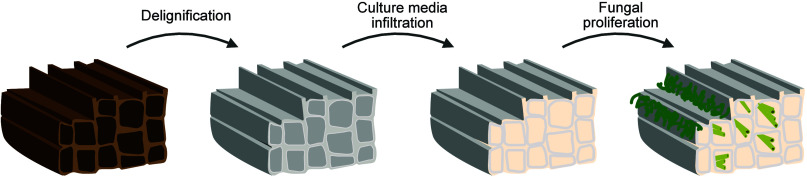
Illustrative overview of wood delignification with color change from brown to gray, media infiltration with color change from gray to beige, and fungal inoculation with color change from beige to green and gray.

### Tuning the Mechanical Properties of Delignified Wood with Agar

We evaluated the mechanical properties of three wood scaffolds, balsa, poplar, and spruce, with and without infiltrated agar. We successfully delignified all three wood types, as confirmed with FTIR (Supplementary Figure S1). The delignified wood scaffolds were infiltrated with culture media containing varying concentrations of agar (0, 0.5, 2, or 4 wt %) (Figure 2). Balsa wood is characterized by fibers, rays, and large vessels, which are typical features of softwood^30^ (Figure 2 b). In contrast, poplar a hardwood consists of large vessels surrounded by small fibers^31^ (Figure 2c). Spruce, another softwood, is distinguished by long tracheid structures^32^ (Figure 2d). The lumina and vessels of all wood scaffolds were filled after the addition of 2 wt % agar (Figure 2e–g). However, some cavities remained unfilled.

**Figure 2:**
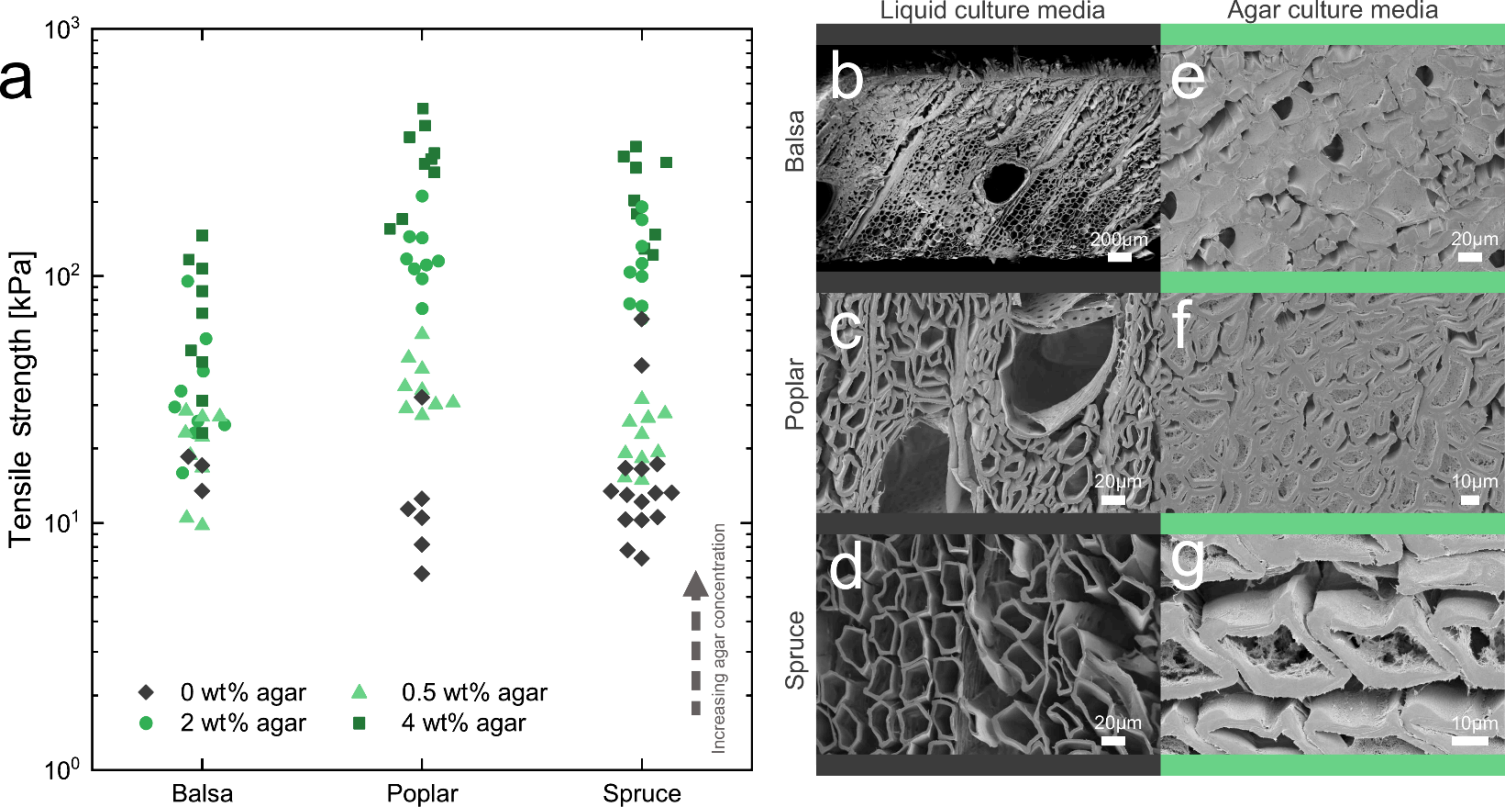
Delignified wood filled with culture media: (a) tensile strength of balsa, poplar, and spruce delignified wood scaffolds with increasing concentrations of agar in the infiltrated media, and (b) to (g) SEM images of the three wood types infiltrated with liquid culture media (left) and 2 wt % agar culture media (right).

Tensile tests revealed that there is no significant effect of the morphology of wood on the mechanical properties (Figure 2a). However, it was expected that, as the density of wood increases, the mechanical properties would also increase.^33,34^ Among the wood types studied, spruce had the highest density, followed by poplar and then balsa (Supplementary Table S1). Balsa wood shows an unexpectedly high mechanical strength in relation to its density.^30^ The density did not correlate directly with the mechanical strength of the delignified wood pieces, most likely highlighting the importance of the fiber morphology effect on tensile strength.

An increase in the agar concentration resulted in an increase in tensile strength across all three wood types. The improvement in mechanical properties after the infiltration with hydrogel was expected.^25^ The tensile strength of composites of wood with agar was highly affected by the choice of wood type and agar concentration (*p* < 0.001 for both factors wood type and agar concentration), indicating that the choice of wood and agar concentration are important factors that affect the mechanical properties of the composites. Since the trends in Young’s modulus were similar to those for tensile strength, we opted to present only the tensile strength data (Supplementary Figure S2).

In summary, delignified wood composites offer great potential for applications requiring mechanical reinforcement, as their mechanical properties can be easily modified. This can be achieved by the addition of polymers, such as agar.

### Anisotropic Fungal Growth Increases Tensile Strength

The delignified wood scaffolds from balsa, poplar, and spruce were infiltrated with liquid culture media, inoculated with either *A. oryzae* or *R. oligosporus*, and incubated. The incubated wood–fungi scaffolds exhibited higher tensile strength and Young’s Modulus in comparison to ungrown control wood scaffolds (Figure 3a,b). The growth of filamentous fungi in the composites increased the mechanical properties of delignified wood (*p* < 0.001 for factor fungi). This can be attributed to the increase in biomass and most likely to the fibrous and chemical nature of mycelium. The cell walls of filamentous fungi are made of chitin, which could contribute to the high mechanical strength.^35,36^ Although fungal growth was somewhat limited, probably due to low oxygen transfer in the structure and restricted spatial growth, the fungi grew along the wood fibers and infiltrated the lumina and cavities (Figure 3e–h). The hyphae exhibited anisotropic growth and were compelled to grow in the direction of fibers, as the used fungal species have low cellulase activity.^37,38^ In contrast, fungi in homogeneous environments such as hydrogel culture media exhibit isotropy and radial growth (Supplementary Figure S3).

**Figure 3:**
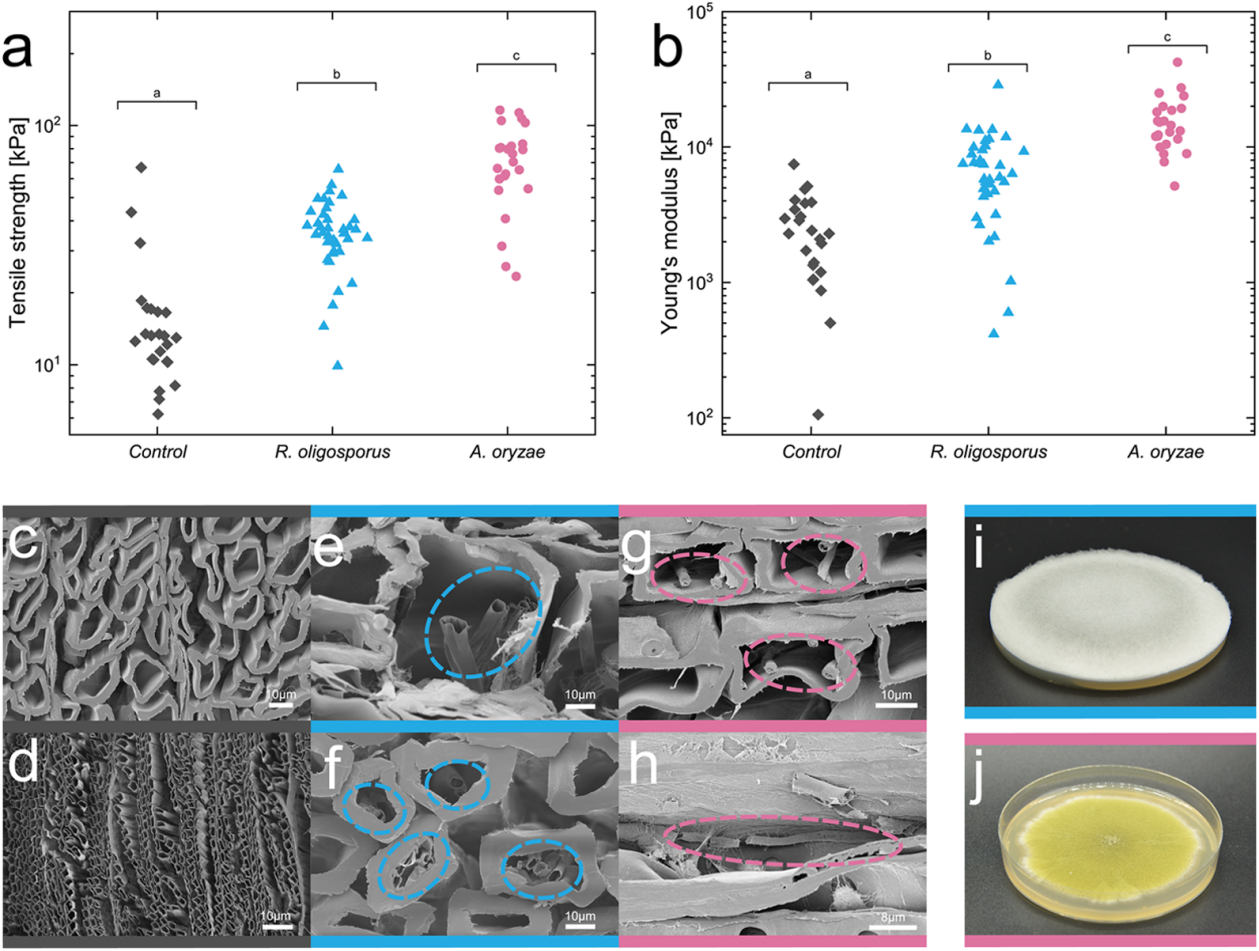
(a) Tensile strength and (b) Young’s modulus of wood–fungi composites inoculated with *R. oligosporus* or *A. oryzae* measured with tensile tests. The delignified wood (balsa, poplar, and spruce) scaffolds were infiltrated with liquid culture medium. (c) and (d) show SEM images of control scaffolds without fungi, and (e) to (h) show SEM images of wood scaffolds after proliferation. (i) shows the growth of *R. oligosporus* and (j) the growth of *A. oryzae* on culture media gelled with 2 wt % agar. Images with blue side marks are from cultures with *R. oligosporus*, images with pink side marks from cultures with *A. oryzae*. Circles indicate fungal growth inside the wood scaffolds.

The increase of tensile strength and Young’s modulus through fungal growth on wood scaffolds is strongly dependent on the cellulase activity of the chosen species. Many filamentous fungi act as saprophytes, degrading organic material including wood.^39^ Previous studies showed a decrease in compressive strength after the proliferation of fungi on cork, due to degradation of the material.^40^ Similarly, Haneef et al.^41^ found that fungal material was higher in mechanical properties if grown on amorphous cellulose, which is harder to digest than crystalline cellulose. The fungal species chosen in our study have a low cellulase activity.^37,38^ Therefore, the fungi did not degrade the material but increased the mechanical properties.

Additionally, there was also a highly significant difference in tensile strength and Young’s modulus between the wood–fungi composites with *A. oryzae* and with *R. oligosporus*. Wood–fungi composites with *A. oryzae* had higher tensile strength and Young’s Modulus compared to composites with *R. oligosporus*. This could be due to the morphology of the mycelium formed by *A. oryzae*. While *A. oryzae* has septate mycelium, *R. oligosporus* is aseptate.^42^ The presence of septa in the hyphae of *A. oryzae* could contribute to its increased mechanical strength.^43^ As a result, *A. oryzae* may have been more effective than *R. oligosporus* in improving the tensile strength and stiffness of wood scaffolds. Moreover, the two fungi exhibit different amounts of aerial and substrate hyphae. *R. oligosporus* grows less dense in fluffy patterns, producing a tall and visible portion of aerial mycelium (Figure 3i). In contrast, the mycelium of *A. oryzae* consists mostly of penetrative hyphae, with its surface covered by conidiophores and a small amount of aerial mycelium (Figure 3j). These morphological differences may also affect the density of the hyphae within the delignified wood scaffolds, ultimately affecting their mechanical properties.

In conclusion, filamentous fungi have significant potential to improve the mechanical properties of wood composite materials. By utilizing delignified wood, we can achieve anisotropic growth patterns in fungi. Choosing an ideal fungal strain is crucial in determining how effectively fungi can strengthen the composite material as degradation can decrease the mechanical properties. Hence, these composites act as suitable scaffolds, providing an ideal environment for anisotropic fungal growth.

### Influence of the Wood Type and Culture Media on Mechanical Properties

The mechanical properties of wood–fungi composites depend on the wood scaffold morphology, the media’s nutrient content, and the microorganism’s selection. As both fungi exhibit similar mechanical properties as a function of wood scaffold morphology and nutrient availability, we will focus on the mechanical properties of wood–fungi composites of *A. oryzae*. The tensile strength of the wood–fungi composites infiltrated with liquid culture media was found to be significantly higher than those of the control samples across all wood types and the wood type was significant (*p* < 0.001 for factor wood type) (Figure 4a). The overall effect of the factor wood on Young’s modulus was identical (Supplementary Figure S4). Among poplar, spruce, and balsa wood, the tensile strength increase was least pronounced in spruce, with an average tensile strength increase from 18.13 to 52.20 kPa. In contrast, the most substantial increase was observed in balsa and poplar wood–fungi composites, which increased from an average tensile strength of 16.36 to 85.18 kPa (balsa) and an average of 13.51 to 78.43 kPa (poplar). This suggests that spruce’s higher density and smaller lumen size may have limited fungal growth compared to balsa and poplar.

**Figure 4:**
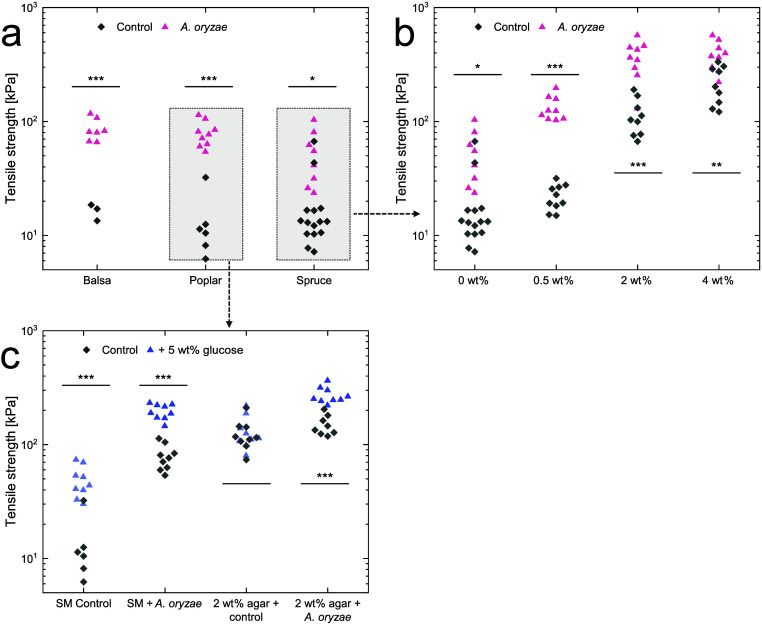
Influence of (a) wood type, (b) agar concentration, and (c) glucose on the tensile strength of wood–fungi composites.

Subsequently, various agar concentrations were incorporated into the malt extract media to elucidate the effect of culture media viscoelasticity on fungal growth within wood–fungi composites. As shown previously, both tensile strength and Young’s modulus increased with increasing agar concentrations in the wood scaffolds with infiltrated agar (see Figure 2). We expected that the tensile strength of fungal-inoculated samples would also increase with higher agar concentrations. This is due to increased fungal elasticity in culture media with higher elasticity.^12^ Nevertheless, fungal growth might be limited due to the media occlusions of the vessels and tracheids, constraining the space for fungal proliferation. Observations from growth assays conducted on agar Petri dishes indicate that variations in agar concentrations did not significantly impact the radial extension rate (Supplementary Figure S5). However, the effect of viscoelasticity on 3D growth has not been fully explored.

The tensile strength of wood scaffolds inoculated with *A. oryzae* increased with increasing agar concentration (Figure 4b); despite the increased filling of the wood cavities, fungal growth was sustained and the factor agar was significant (*p* < 0.001 for factor agar concentration). Similar results were found for the Young’s modulus (Supplementary Figure S6). The least significant difference was measured between the liquid control and the fungal samples, whereas the most pronounced difference occurred at agar concentrations of 0.5 and 2 wt %. This suggests that the increasing viscoelasticity of the culture media could lead to an increase in tensile strength.^12^ Additionally, we propose that 4 wt % agar in the culture media contributed to a reduction in penetration depth, as viscoelastic materials typically show lower penetration depth compared to liquid material.^42,44–46^

To further improve fungal growth and, therefore, tensile strength, we added glucose to foster fungal proliferation. To predict fungal growth rates, we quantified the fungal propagation rates by measuring the colony diameter on Petri dishes containing various nutrient adjustments (Supplementary Figure S7). The addition of 5 wt % glucose to malt extract media significantly increased propagation speed. Subsequently, we adjusted the culture media for wood scaffold infiltration with 5 wt % glucose in both liquid- and agar-containing media and inoculated with *A. oryzae*. The addition of glucose significantly increased tensile strength and Young’s modulus (Figure 4c and Supplementary Figure S8). We suggest that the faster growth rate caused by glucose led to more biomass in the composites, which enhanced their mechanical strength.^10^ Additionally, we hypothesize that glucose may function as a lubricant within the composite, increasing the mechanical properties as the control samples without fungi showed improved mechanical properties.^1,47^

The wood–fungi composites can be precisely engineered to promote fungal growth and thus increase their mechanical strength. As a result, these composites represent a promising option for developing finely tuned, functional, and sustainably engineered living materials.

### Toward Engineered Living Material

Mycelium and mycelium-based composites have emerged as a distinctive category of materials that occupy an intermediate position between natural cellular material and natural elastomers.^1,48^ In our work, we obtained wood–fungi composites with mechanical properties similar to those of other mycelium composites (Figure 5a). The composite material from delignified wood and culture media had Young’s moduli of around 10^–3^ GPa (Figure 5b). Following fungal proliferation, Young’s modulus increased by up to 1.5 decades. By improving nutrient availability with the addition of glucose, the Young’s Modulus increased even further by 1 decade. The desired mechanical strength can be fine-tuned with the proper selection of wood, fungi, hydrogel concentration, and nutrients.

**Figure 5:**
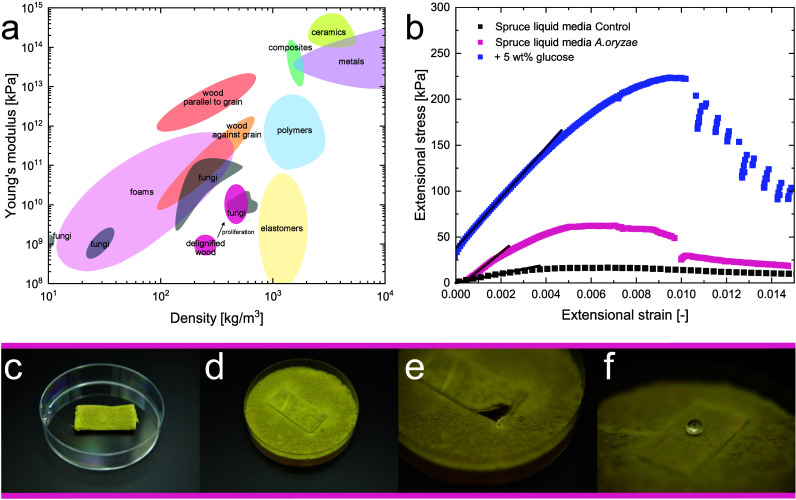
(a) Young’s modulus as a function of the density of various materials, adapted from Wegst and Ashby^50^ and Gibson et al.^51^ with literature data for fungal material,^1,11,52,53^ (b) stress–strain curve of selected measurements with Young’s modulus, (c) wood–fungi composite after proliferation, (d) and (e) wood–fungi composite 5 days after reincubation on malt extract media, and (f) water drop on the wood–fungi composite.

A key property of engineered living materials is their ability to remain biologically active to utilize the adaptive properties and dynamic responsiveness of fungi.^2,10^ Here, we demonstrate that our living material remained viable after 14 days of incubation. To confirm their livingness, the samples were reincubated on malt extract media. Within 5 days, the fungus had completely overgrown the Petri dish (Figure 5c–e). To improve the longevity of such living materials, we could integrate spores into the dried delignified wood samples. After hydration, such spores could grow in the scaffolds, creating an on-demand stiffness and reactivity in the material. Such a step would form a responsive and living material that, after growth, would also be water-repellent through the hydrophobic coating of the fungal surface by hydrophobins^49^ (Figure 5 f).

## Conclusion

Delignified wood can serve as an excellent scaffold for fungal proliferation, as fungi grow along the wood fiber structure, demonstrating anisotropic growth. The tensile strength and Young’s modulus significantly increased after fungal proliferation, showcasing the potential of these composite materials in various applications. The enhancement of mechanical properties highly depends on the type of delignified wood, nutrient concentration, and selected fungal species. Both fungi did not degrade the wood but instead enhanced its mechanical properties. Composites with *Aspergillus oryzae* exhibited higher tensile strength than those with *Rhizopus oligosporus*, likely due to the septate and penetrative mycelium formed by *Aspergillus oryzae*. This characteristic can be leveraged to create novel engineered living materials with high tensile strength due to natural growth patterns. Ultimately, this work underscores the significance of filamentous fungi in enhancing mechanical strength in composite materials, the suitability of delignified wood as a scaffold, and the tunability of both fungal growth and delignified wood material. The resulting composite could be utilized as a building or packaging material.

## Supplementary Material



## Notes

SEM imaging, density of woods in Table S1, FTIR results in Figure S1, Young’s modulus data in Figures S2, S4, S6, and S8, images of fungal growth in Figure S3, and propagation speed in Figures S5 and S7 (PDF)
