# Supplementary material for: Anisotropic Growth of Filamentous Fungi in Wood Hydrogel Composites Increases Mechanical Properties
Source: ACS Appl Bio Mater. 2025 Jun 6;8(6):5024–31. doi: 10.1021/acsabm.5c00374 (PMC12175164; doi:10.1021/acsabm.5c00374)
Supplement: Supplementary file 1 [file mt5c00374_si_001.pdf]

Supporting Information: Anisotropic growth of filamentous fungi in wood hydrogel composites increases mechanical properties

Ciatta Wobill, Ziyu Zhang, Peter Fischer, and Patrick Rühs*

Institute of Food, Nutrition and Health, ETH Zürich, 8092 Zürich, Switzerland

E-mail: patrick.ruehs@hest.ethz.ch

Phone: +41 44 632 36 68

SEM Imaging

Sample processing was done in a PELCO BioWave, Pro+ microwave system (Ted Pella, USA), following a microwave-assisted fixation and dehydration procedure. Small rectangular test pieces about 1 mm wide and 10 mm long were cut out of the wooden plates. Fixation was done in 2.5 % glutaraldehyde/ 2 % paraformaldehyde in cacodylate buffer. After washing the samples were postfixed in 1 % OsO4 in bidistilled water, washed again, and dehydrated in a graded series of ethanol (25 %, 50 %, 75 %, 90 %, 98 %, and three times 100 %) on ice followed by critical point drying out of dry ethanol (CPD 931, Tousimis, USA). Three slices of the dried wooden piece were cross-cut with a blade at the end, where the interaction with the fungi started, and mounted on SEM aluminum stubs with conductive carbon paint (Plano, Germany). Rotary sputter-coating with 6 nm of platinum/palladium (CCU-10, Safematic, Switzerland) was done after drying. For FE-SEM-imaging at an accelerating voltage of 2 kV (Merlin, Zeiss, Germany), SE-inlens and Everhart-Thornley (ET)

SE-signals were recorded at a working distance of around 5 mm.

Sample processing was done in a PELCO BioWave, Pro+ microwave system (Ted Pella, USA), following a microwave-assisted fixation and dehydration procedure. Small rectangular test pieces about 1 mm wide and 10 mm long were cut out of the wooden plates. Fixation was done in 2.5 % glutaraldehyde/ 2 % paraformaldehyde in cacodylate buffer. After washing the samples were postfixed in 1 % OsO4 in bidistilled water, washed again, and dehydrated in a graded series of ethanol (25 %, 50 %, 75 %, 90 %, 98 %, and three times 100 %) on ice followed by critical point drying out of dry ethanol (CPD 931, Tousimis, USA). Three slices of the dried wooden piece were cross-cut with a blade at the end, where the interaction with the fungi started, and mounted on SEM aluminum stubs with conductive carbon paint (Plano, Germany). Rotary sputter-coating with 6 nm of platinum/palladium (CCU-10, Safematic, Switzerland) was done after drying. For FE-SEM-imaging at an accelerating voltage of 2 kV (Merlin, Zeiss, Germany), SE-inlens and Everhart-Thornley (ET) SE-signals were recorded at a working distance of around 5 mm.

Table S1: Density of wood

Wood type	Density [kg/m^3]
Balsa	225.64
Poplar	361.00
Spruce	371.45

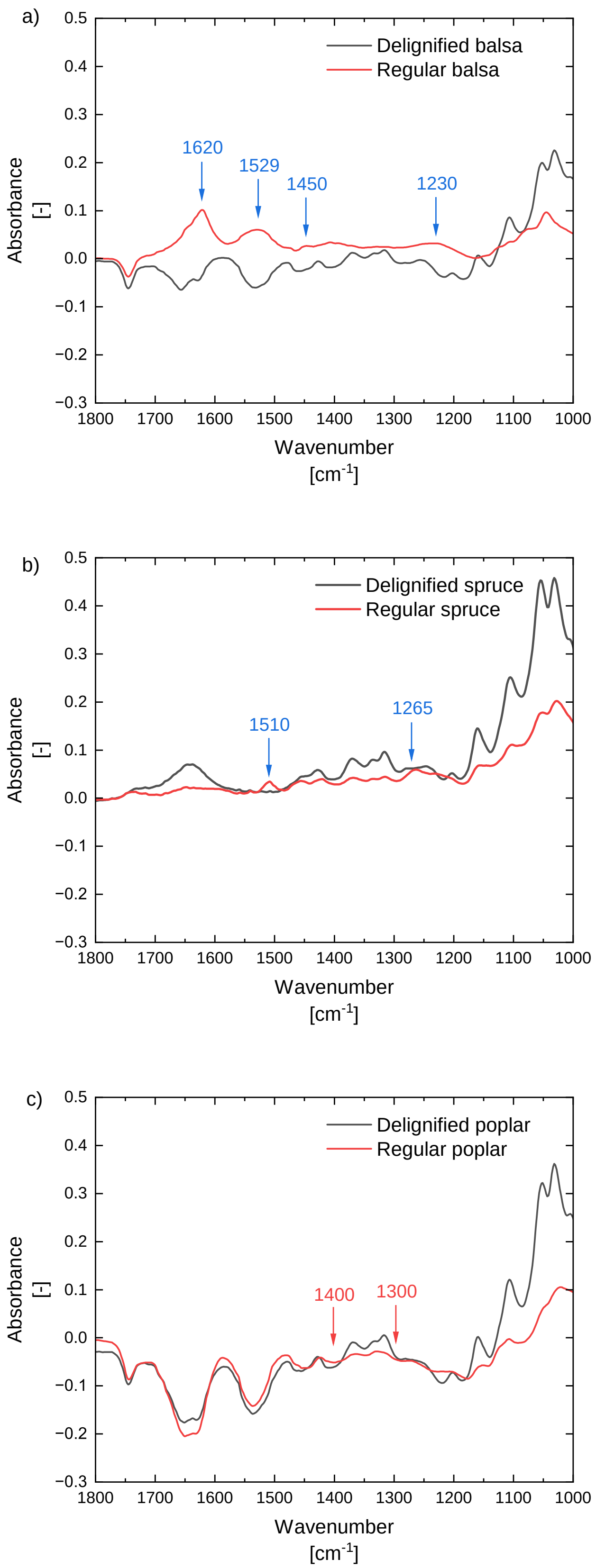

Figure S1: FTIR spectra before and after delignification of (a) balsa, (b) spruce, and (c) poplar.

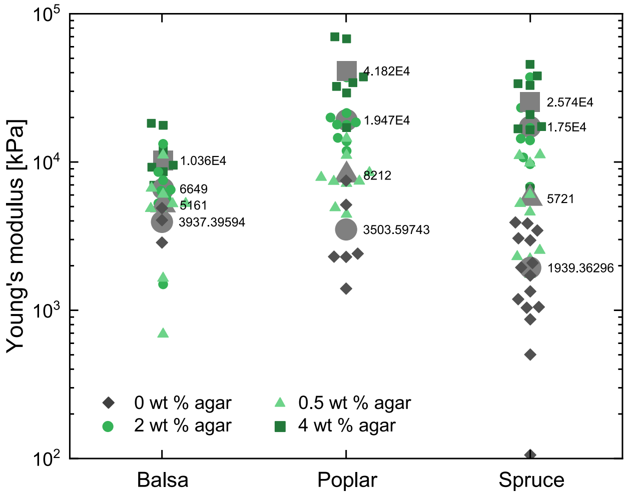

Figure S2: Young's modulus of delignified wood filled with culture media of increasing concentrations of agar.

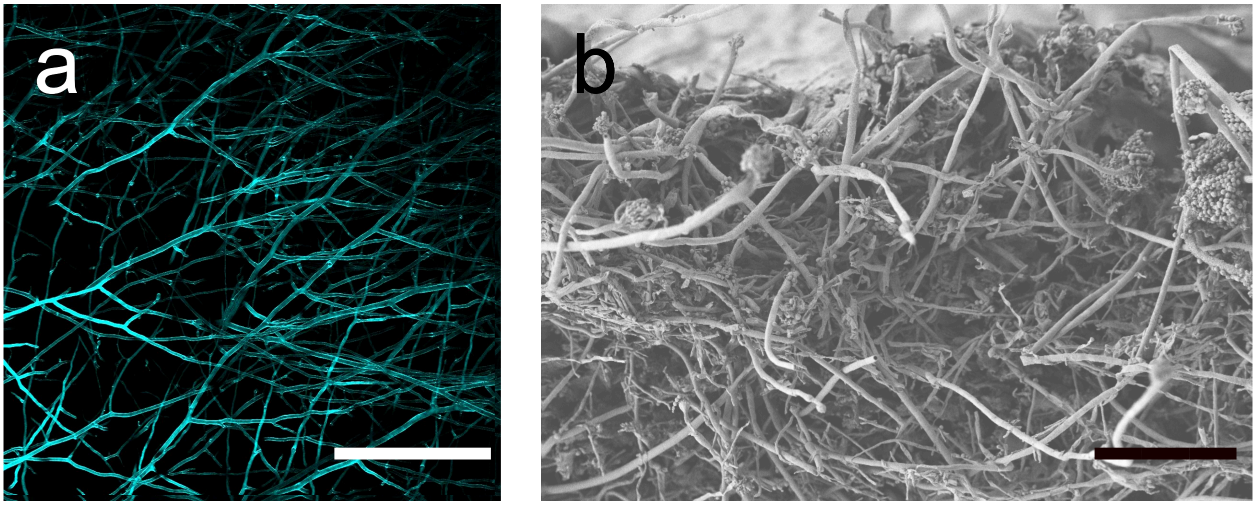

Figure S3: Isotropic growth of filamentous fungi: (a) confocal microscopy image of fungal growth in hydrogel (scale bar = 400 μm) and (b) SEM image of surface growth on wood-fungi composite (scalebar = 120 μm

)

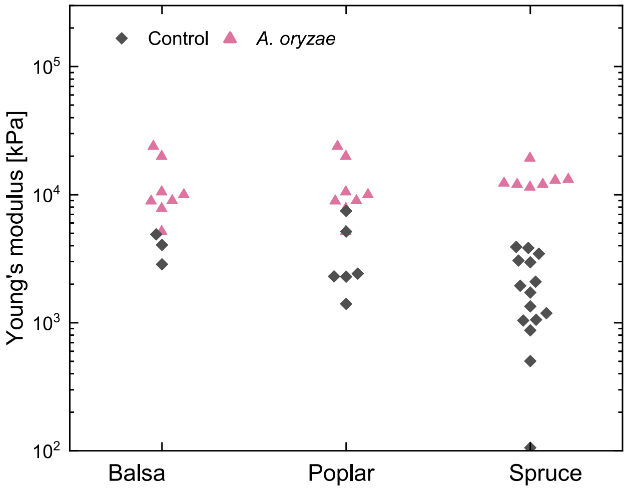

Figure S4: Influence of wood type on Young's modulus of delignified wood - fungal composites. Factor wood is significant ($p < 0.001$).

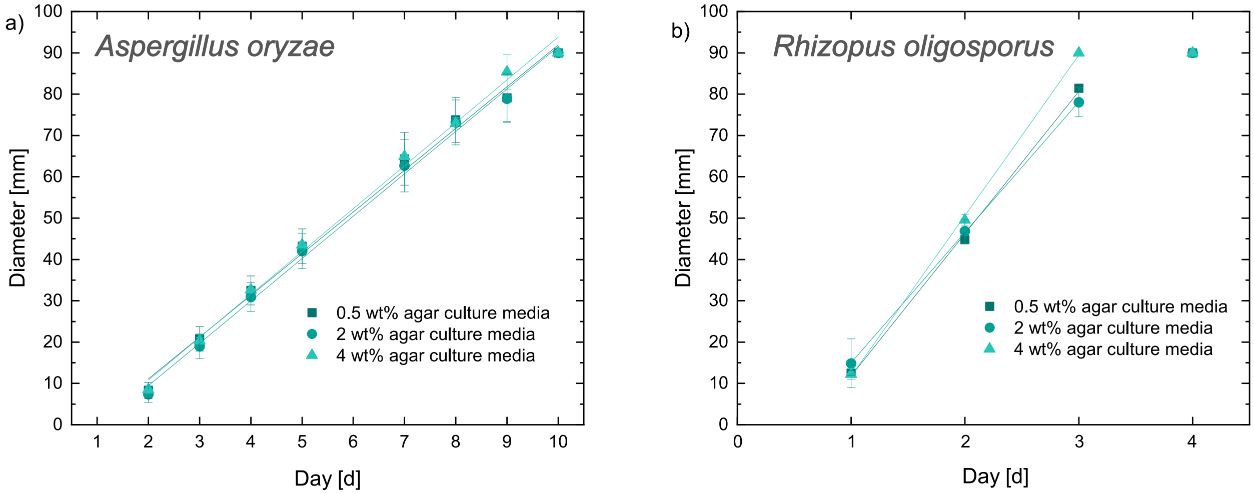

Figure S5: Propagation speed in agar malt extract media of (a) *Aspergillus oryzae* and (b) *Rhizopus oligosporus*.

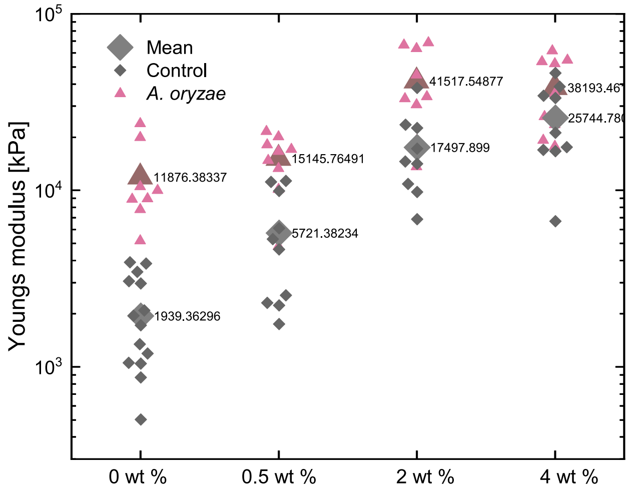

Figure S6: Influence of agar concentration on Young's modulus of delignified wood-fungal composites.

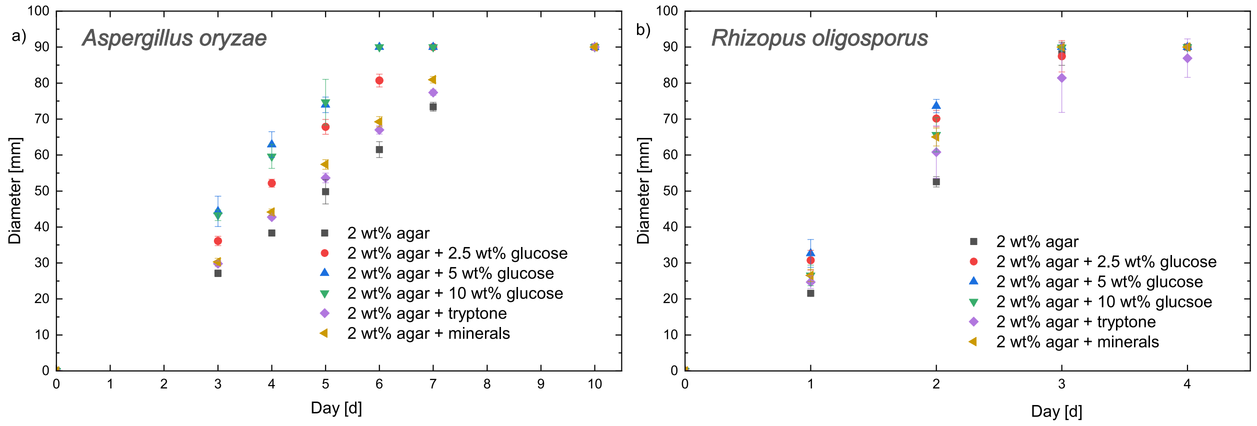

Figure S7: Propagation speed in agar malt extract media with additional nutrients of (a) *Aspergillus oryzae* and (b) *Rhizopus oligosporus*.

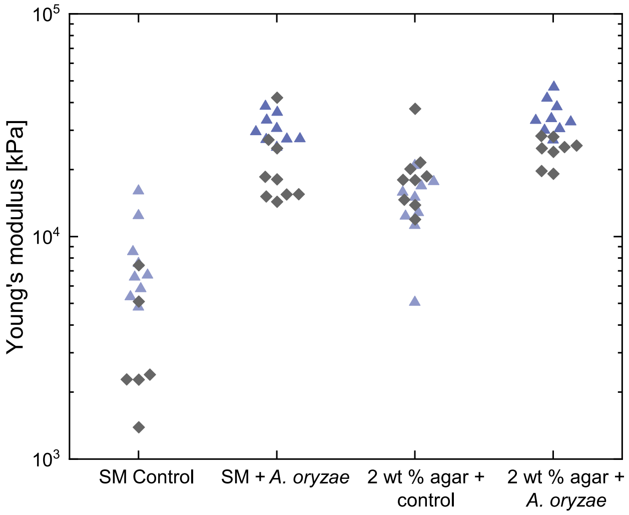

Figure S8: Influence of glucose on Young's modulus of delignified wood - fungal composites.
